# Endovascular Management of Mucormycotic Aneurysms of the Internal Carotid Artery in Post-COVID-19 Patients

**DOI:** 10.7759/cureus.20812

**Published:** 2021-12-29

**Authors:** Sibasankar Dalai, Aravind Varma Datla, Abhilash A Francis, Naveen K Dannana, Hameed Parappil

**Affiliations:** 1 Interventional Neuroradiology, Medicover Hospitals, Visakhapatnam, IND; 2 Internal Medicine, Medicover Hospitals, Visakhapatnam, IND; 3 ENT, Head and Neck Surgery, Badr Al Samaa Hospital, Al Farwaniyah, KWT; 4 ENT, Head and Neck Surgery, Apollo Hospitals, Visakhapatnam, IND; 5 ENT, Badr Al Samaa Hospital, Al Farwaniyah, KWT

**Keywords:** rhinocerebral mucormycosis, epistaxis, flow diverter, parent artery sacrifice, aneurysm coiling, antifungal drugs, mycotic aneurysms, endovascular procedures, post-covid-19 complications, covid-19

## Abstract

The repercussions of the coronavirus disease 2019 (COVID-19) are being felt throughout the world to this day. India is one such country ravaged by the second wave of the pandemic. Here, we report two cases of post-COVID-19 mucormycotic aneurysm of the internal carotid artery (ICA), which we believe are the first of their kind in the reported medical literature. A nasopharyngeal swab for reverse-transcriptase polymerase chain reaction of the severe acute respiratory syndrome coronavirus 2 was positive for both cases. After recovering from COVID-19, both patients developed signs and symptoms suggestive of mucormycosis, which were confirmed by a fungal smear. They were managed with liposomal amphotericin B (LAmB) and other adjunctive medicines. The first patient developed a massive bout of epistaxis during surgical debulking of his fungal mass. He underwent anterior nasal packing and emergency digital subtraction angiogram which revealed an aneurysm of the right ICA which was treated by coiling of the aneurysm and parent artery occlusion of the right ICA. The second patient had a history of post-COVID-19 mucormycosis which was managed by LAmB, surgical debulking, and posaconazole. He was not in regular follow-up and did not fully complete his antifungal therapy. Later, he presented with recurrent episodes of epistaxis followed by a massive bout of bleeding from both nostrils which upon evaluation revealed a thin-walled aneurysm of the left cavernous ICA. He was treated with flow diversion and coiling. Both patients responded well and the aneurysms were successfully excluded from the circulation. Their follow-ups were uneventful.

## Introduction

The 2019 novel coronavirus disease (COVID-19), initially identified in Wuhan, Hubei, China, in December 2019, has spread exponentially to around 200 countries. On February 12, 2020, the World Health Organization (WHO) declared COVID-19 a public health emergency of international concern [1]. The WHO declared COVID-19 a global pandemic in March 2020 [2]. India is one of the most affected nations by the pandemic.

As the pandemic continuously evolves, accompanied by a rapid surge in cases, many potential complications are increasingly recognized, including susceptibility to secondary infections [2,3]. Severe COVID-19 is associated with cytokine storm and immune dysregulation, necessitating the use of immunomodulators. This has led to an increasing number of reports of mucormycosis, popularly dubbed as the “black fungus” in post-COVID-19 patients [4].

Agents of mucormycosis have the potential for direct invasion through tissue planes. This proclivity for aggressive invasion, combined with a disposition to colonize the respiratory sinuses, allows these fungi to rapidly access the intracranial structures. The sphenoid sinus, which is located within the sphenoid bone, is separated from the cavernous sinus and the internal carotid artery (ICA) by only a thin layer of the lateral wall of the sphenoid bone leading to mycotic aneurysms of the ICA [5].

The main reason for invasive fungal infections can be the impairment of innate defense mechanisms, such as ciliary clearance, and the lack of sufficient lymphatic immune response against the fungal invasion which can be seen during the pathophysiologic progression of immune deregulation in the COVID-19 phenotype [6].

Here, we report the cases of two patients who developed mucormycotic aneurysms of the ICA after recovering from COVID-19, which we believe are the first of their kind in the reported medical literature.

## Case presentation

Case 1

**Figure 1:**
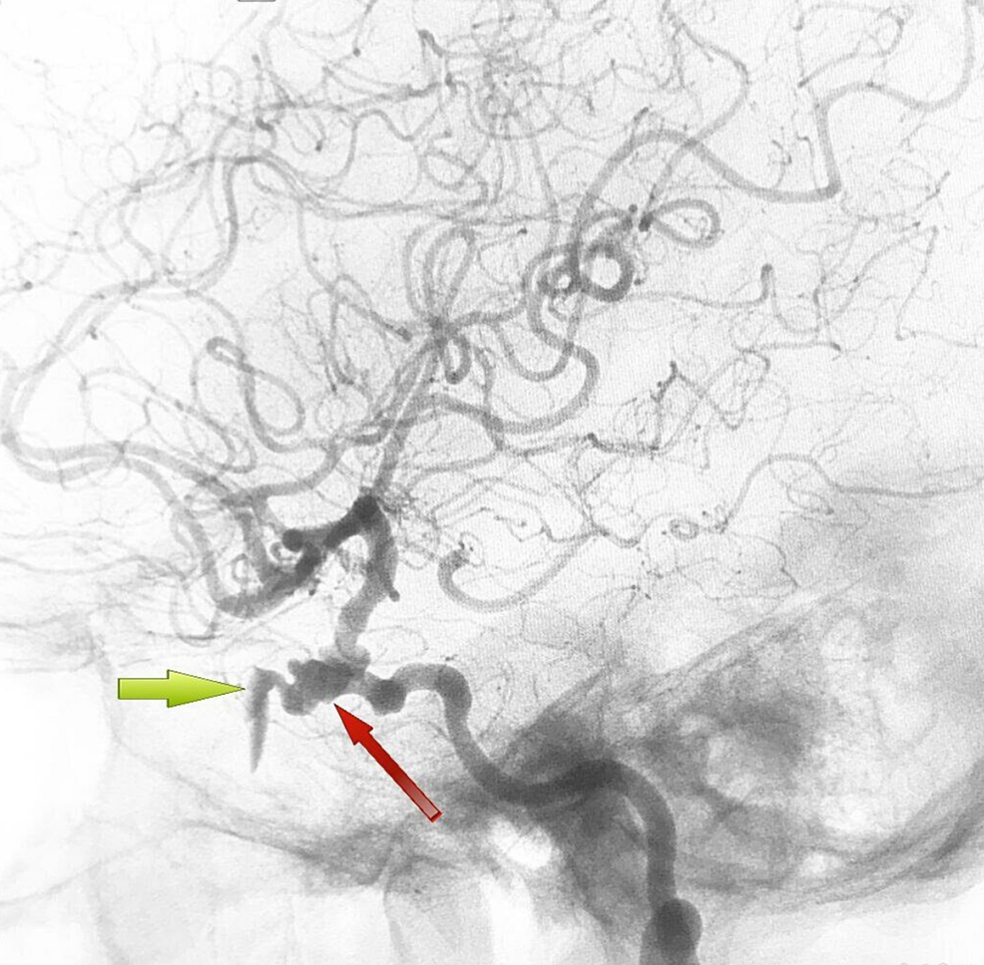
DSA lateral view showing an aneurysm of the right ICA (red arrow) along with the site of the aneurysmal rupture (green arrow). DSA: digital subtraction angiogram; ICA: internal carotid artery

A 53-year-old male presented to his local hospital with a five-day history of fever, generalized weakness, and myalgia. One day prior, he had developed breathlessness and tachypnea. He was a known diabetic and hypertensive on treatment with oral hypoglycemic agents and oral antihypertensive therapy. A nasopharyngeal swab for reverse-transcriptase polymerase chain reaction (RT-PCR) of the severe acute respiratory syndrome coronavirus 2 (SARS-CoV-2) was positive. He was managed with intravenous (IV) antibiotics, high-dose steroids, remdesivir, subcutaneous enoxaparin, and oxygen inhalation, according to the acceptable local norms at the time. Due to his deteriorating oxygen saturation, he was placed on high-flow nasal oxygen (HFNO) for two days, followed by non-invasive ventilation (NIV) for seven days. He was discharged after 14 days of hospitalization. One month after his initial discharge, the patient complained of pain and swelling in the paranasal areas for three days and was subsequently diagnosed as a case of rhinocerebral mucormycosis. He was immediately started on IV liposomal amphotericin B (LAmB). During the surgical debridement of the necrotic tissue, he had a sudden bout of severe epistaxis. The patient went into hypovolemic shock due to massive blood loss, the source of which could not be identified. He was intubated, appropriate volume replacement therapy was initiated, and anterior nasal packing was done with Merocel nasal tampons. The patient was then shifted to the department of interventional neuroradiology for further management. An emergency digital subtraction angiogram (DSA) revealed a right ICA aneurysm measuring 8.8 mm in its largest diameter and medially directed (Figure 1).

Procedure

**Figure 2:**
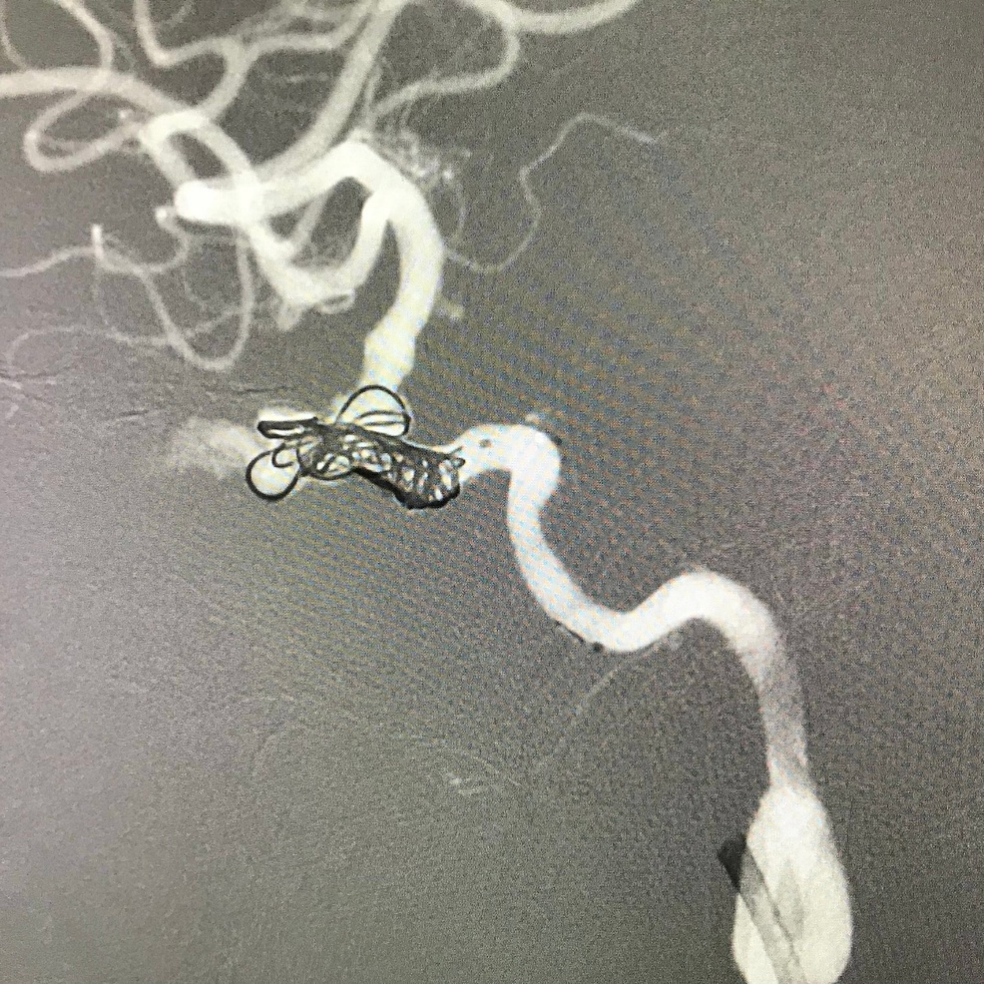
Deposition of the coils within the ICA and the aneurysmal sac under roadmap guidance. ICA: internal carotid artery

The procedure was performed under general anesthesia. Under aseptic conditions, a 6-F short sheath was placed in the right common femoral artery (CFA). The right ICA was canulated using a 4-F Head Hunter diagnostic catheter (Merit OEM, South Jordan, UT, USA), which was exchanged for a 0.035 Amplatz super-stiff wire (Boston Scientific, Marlborough, MA, USA). A 7-F long sheath was placed from the groin into the right common carotid artery (CCA). A 6.4-F distal access catheter (DAC; Stryker Neurovascular, Fremont, CA, USA) was placed in the 7-F long sheath at the level of origin of the aneurysm. An SL10 microcathter and Synchro 0.014 wire (Stryker Neurovascular, Fremont, CA, USA) were navigated in the DAC and placed in the aneurysm. Detachable platinum coils were serially deployed into the aneurysm sac and the parent artery (Figure 2).

**Figure 3:**
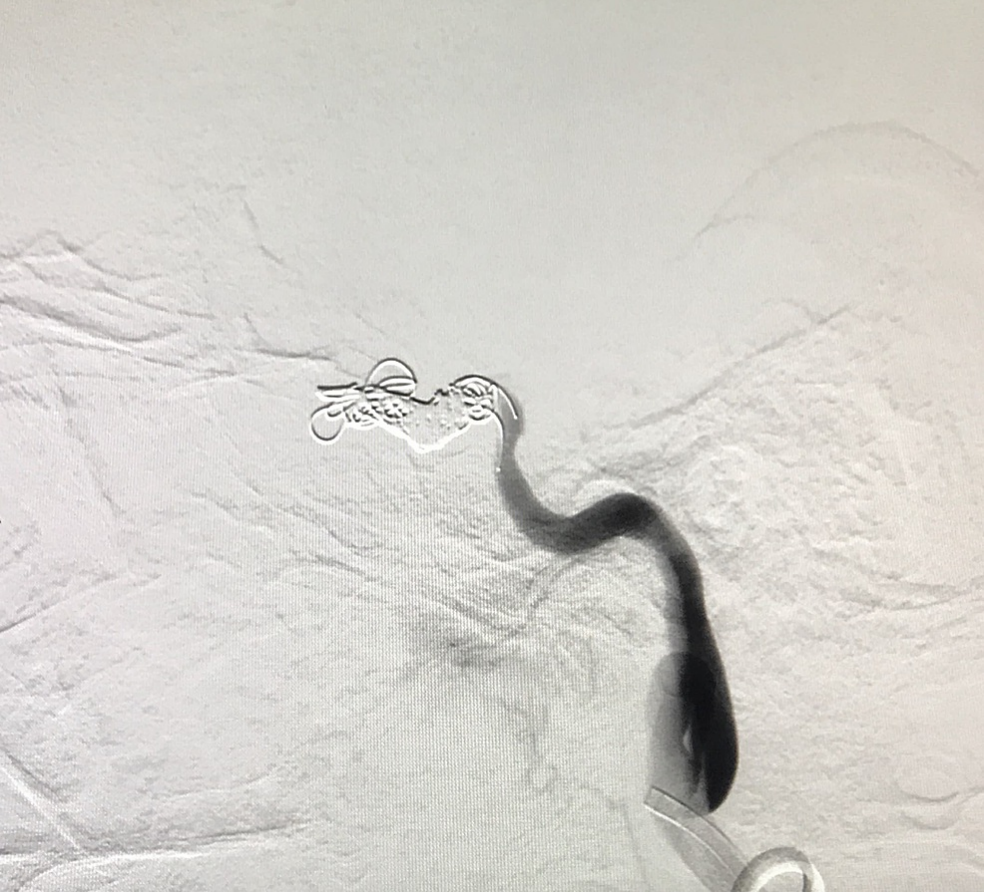
Check angiogram lateral view demonstrating cessation of bleeding from the aneurysm; complete obliteration of the aneurysm with cessation of flow within the ICA and its branches distal to the occlusion. ICA: internal carotid artery

A total of five coils were used to obliterate the aneurysm and occlude the ICA. A check angiogram revealed complete cessation of blood flow within the aneurysm, the ICA, and its territories distal to the occlusion (Figure 3).

**Figure 4:**
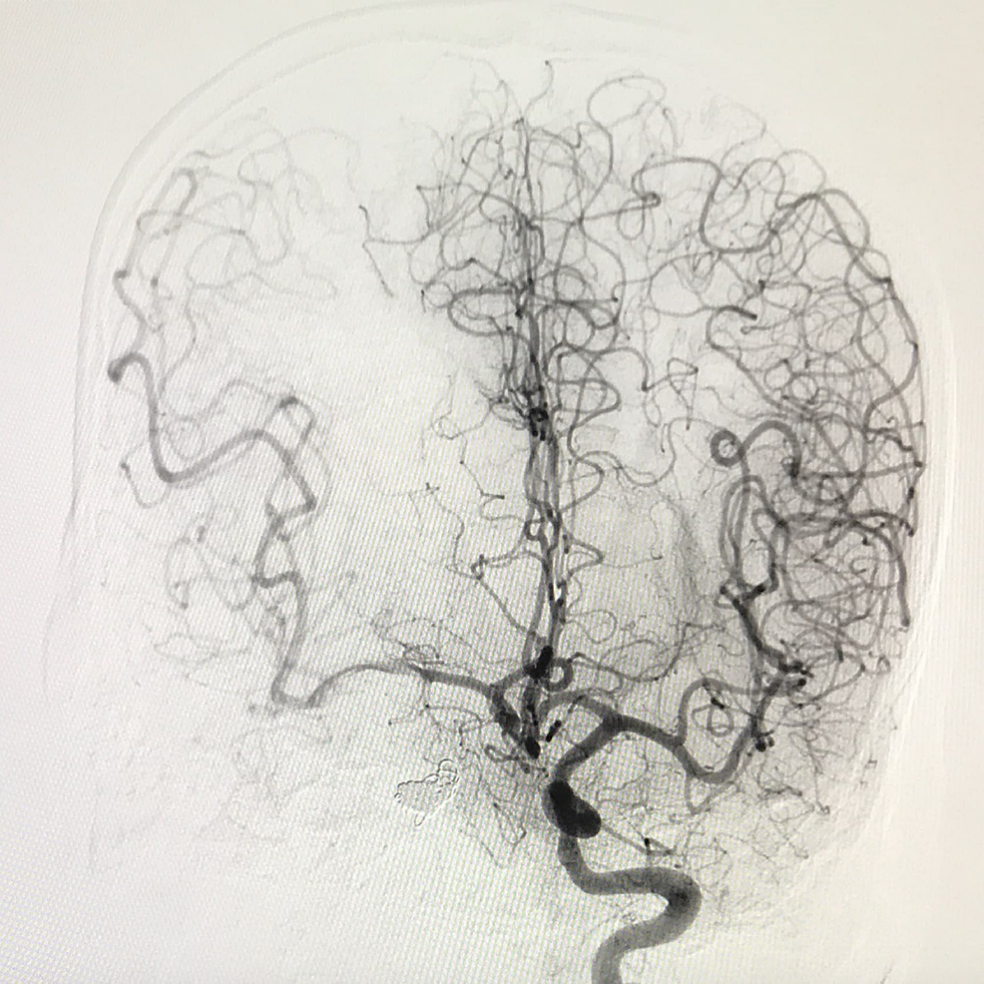
The final post-procedure angiogram demonstrating adequate perfusion of the right cerebral hemisphere from the left-sided cerebral vasculature.

The procedure was well-tolerated by the patient. A final post-procedure angiogram demonstrated adequate perfusion of the right cerebral hemisphere from the left cerebral vasculature (Figure 4).

There were no thromboembolic complications. After five days, the patient was transferred back to his local hospital for further management and to complete his antifungal therapy. At his sixth-month follow-up, the patient had no other significant complaints or recurrence of nasal bleeding.

Case 2

**Figure 5:**
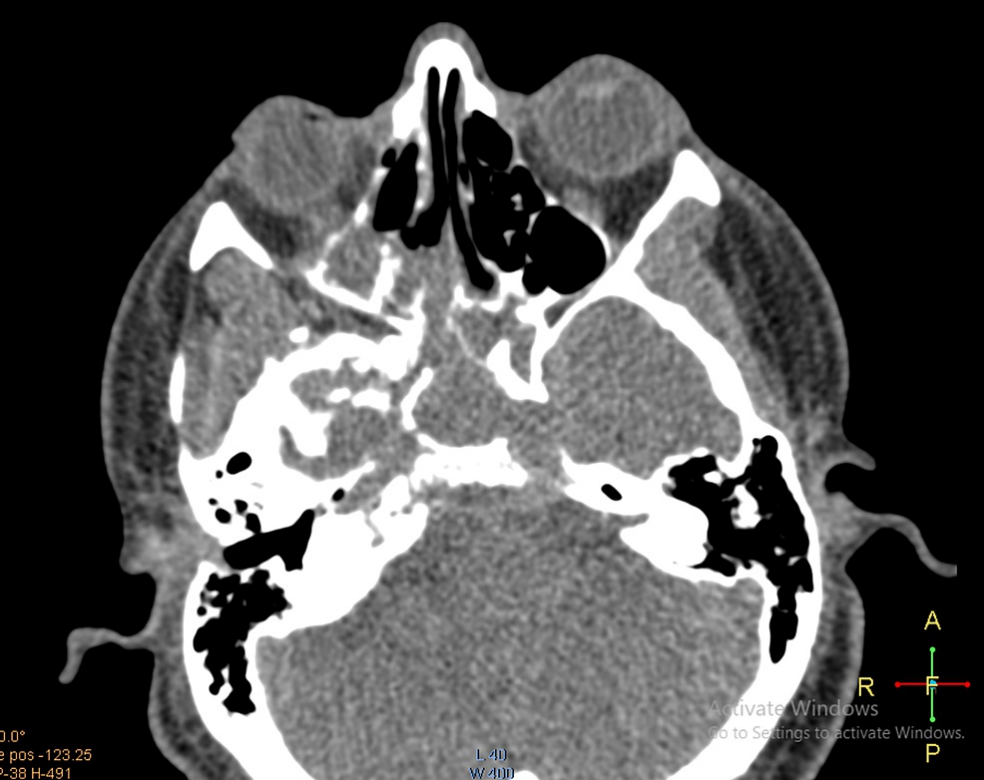
CT of the head axial view showing invasive, left-sided fungal sinusitis. CT: computerized tomography

**Figure 6:**
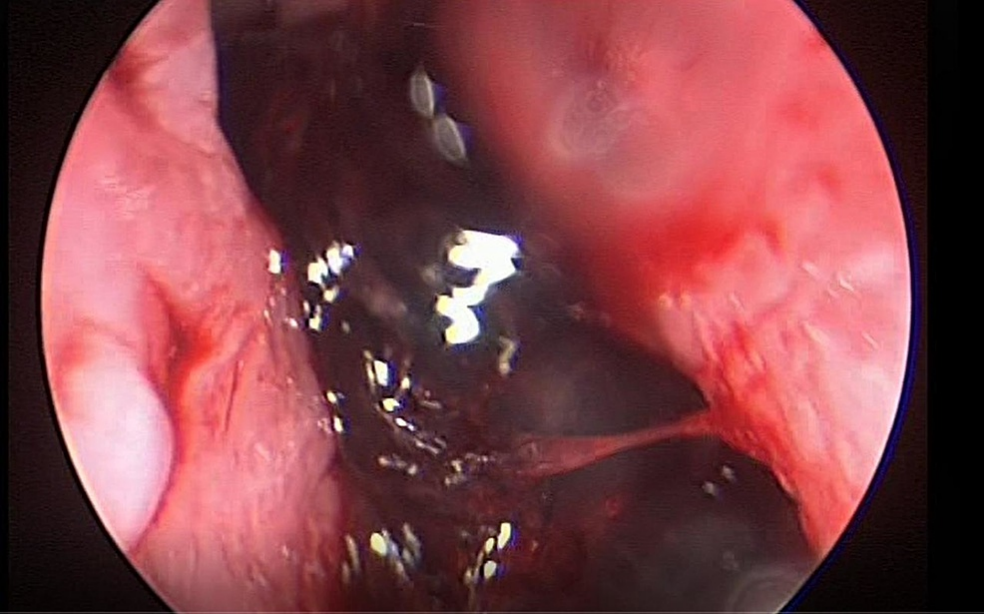
Direct nasal endoscopy revealing a large pulsating clot in the nasal cavity.

A 28-year-old male with no prior comorbidities developed moderately severe COVID-19 four months back, for which he was hospitalized and treated with oxygen inhalation, high-dose systemic steroids, broad-spectrum antibiotics, and remdesivir. Within a week of discharge, he presented to the emergency department with recurrent bouts of nasal bleeding, foul-smelling nasal discharge, retro-orbital pain, and diplopia. Previous nasal bleeds were managed conservatively at his local hospital. The latest bleed was severe, warranting a higher care center visit. Computerized tomography (CT) showed invasive sinusitis (Figure 5), and a fungal smear was positive for Mucorales species. He underwent immediate endoscopic debridement and was started on IV LAmB followed by oral posaconazole 800 mg/day for six weeks. Follow-up magnetic resonance imaging (MRI) of the craniofacial area revealed near resolution with minimal residual disease in the basisphenoid with right cavernous sinus thrombosis. Due to work constraints, he moved out of his hometown, was under irregular follow-up, and stopped posaconazole. Furthermore, he had two episodes of minor nasal bleeding, which were managed conservatively. Later, he presented with a severe bout of uncontrollable nasal bleeding from both nostrils. A direct nasal endoscopic evaluation showed thick blood clots in the right maxillary sinus with a well-healed cavity (Figure 6).

**Figure 7:**
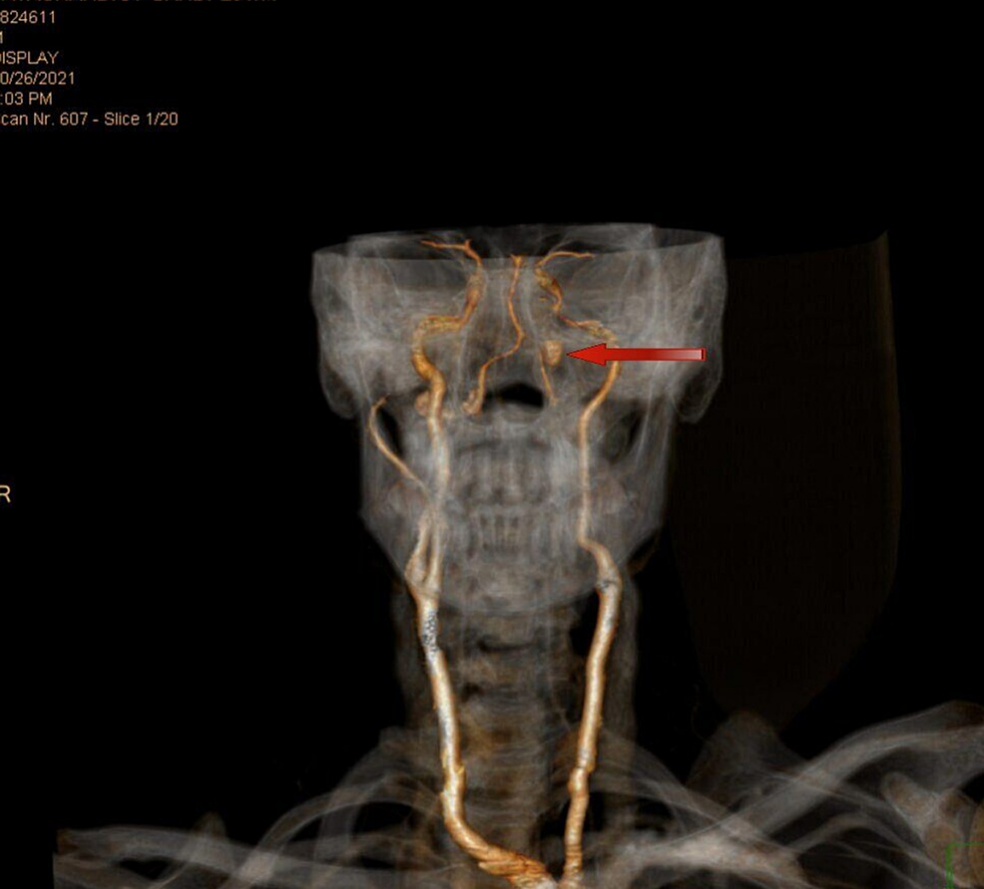
CT angiogram anterior view demonstrating a thin-walled aneurysm (red arrow) in the cavernous segment of the left ICA. CT: computerized tomography; ICA: internal carotid artery

The clots were extending into the sphenoid cavity (left > right), which was exposed due to previous surgical debridement for mucormycosis. No foci of active bleeding were seen. A medicated gel foam was kept in the sphenoid cavity. Considering the history of angioinvasive fungal infection, a CT angiogram was done, which revealed a thin-walled aneurysm in the left cavernous segment of the ICA (Figure 7).

**Figure 8:**
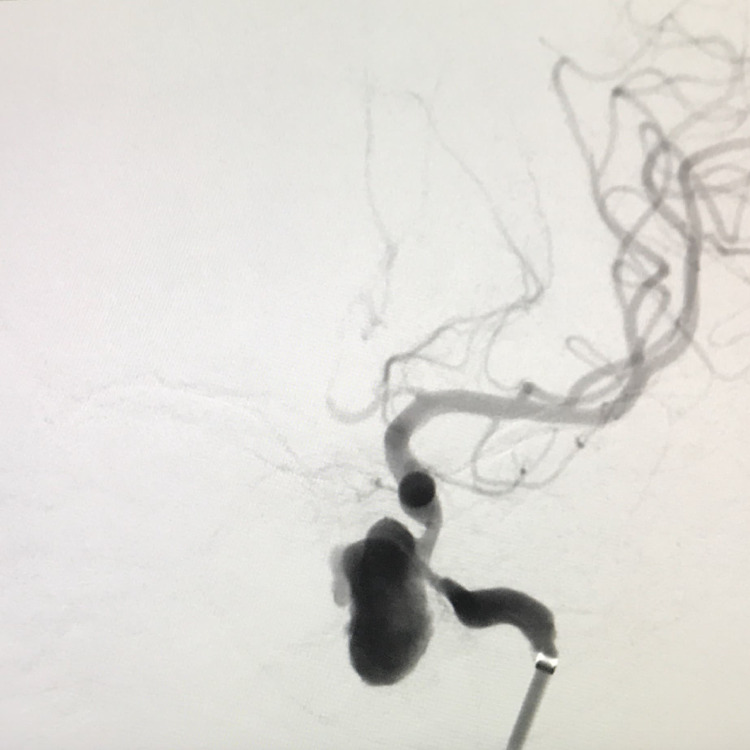
DSA showing a large wide-necked aneurysm of the left ICA. DSA: digital subtraction angiogram; ICA: internal carotid artery

The patient was urgently referred to the department of interventional neuroradiology for further management. A subsequent DSA demonstrated a large aneurysm of the cavernous left ICA, measuring 14 mm in its largest diameter, which was anteromedially directed (Figure 8).

Procedure

**Figure 9:**
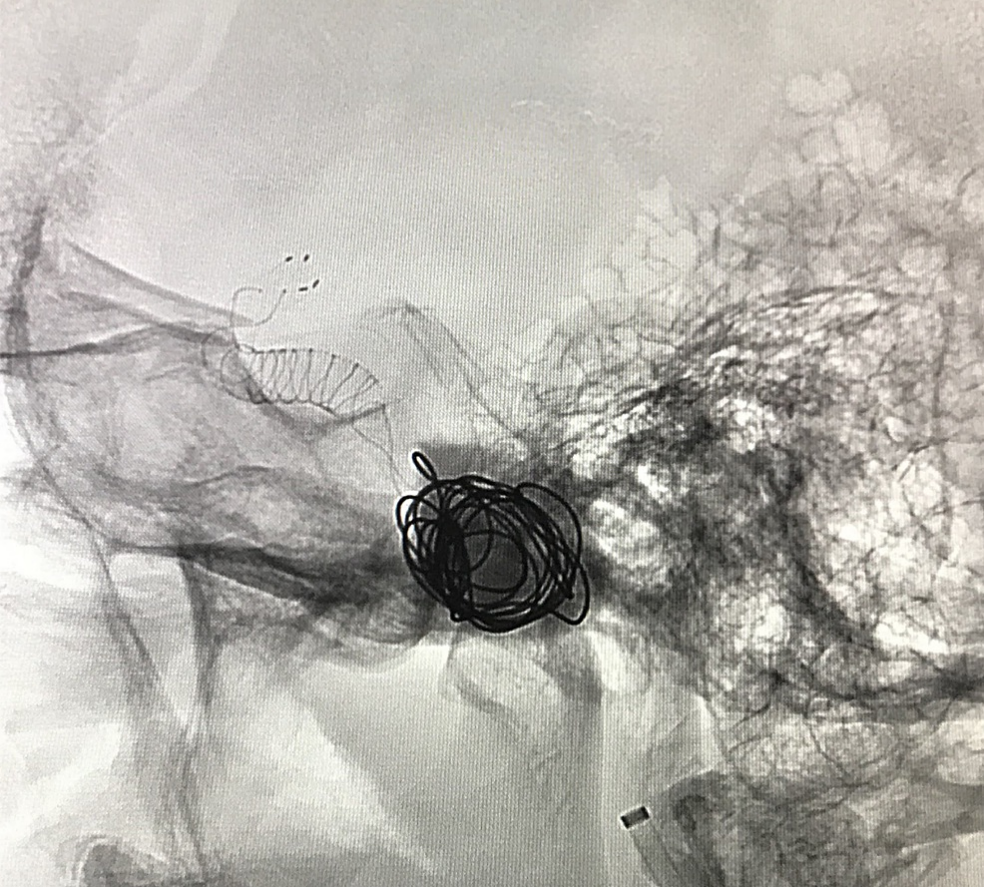
DSA lateral view showing the placement of the flow diverter and coils. DSA: digital subtraction angiogram

The procedure was performed under general anesthesia. As explained above, a 6.4-F DAC was placed proximal to the aneurysm. Headway Duo microcatheter 150 cm (Microvention, Aliso Viejo, CA, USA) was placed inside the aneurysm. Headway 27 microcatheter (Microvention, Aliso Viejo, CA, USA) was navigated into the ICA across the aneurysm, and its distal end was placed at the M1 segment of the left middle cerebral artery (MCA). A FRED 4.5 × 30mm, flow diverter (Microvention, Aliso Viejo, CA, USA) was placed into the ICA across the aneurysm, completely covering its neck. Four coils were serially deployed into the aneurysmal sac through the Headway Duo microcatheter (Figure 9).

**Figure 10:**
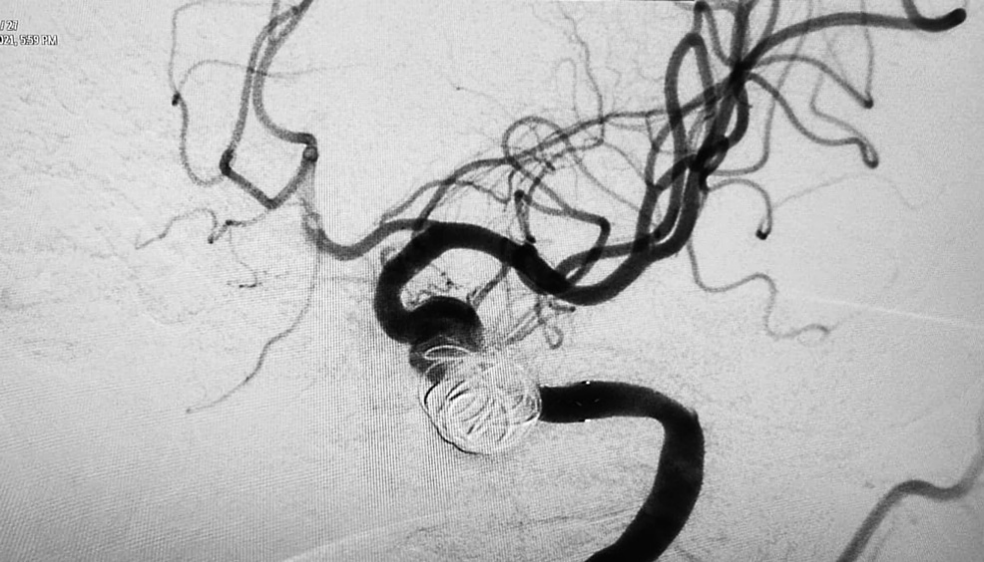
The final angiogram showing the exclusion of the aneurysm from circulation and a near-normal cerebral parenchymogram post-procedure.

A check angiogram demonstrated significant stagnation of contrast within the aneurysm. Both the microcatheters were retrieved, and a final angiogram done after 30 minutes of observation demonstrated near-total exclusion of the aneurysm from the circulation with normal ICA flow and near-normal cerebral parenchymogram (Figure 10).

He was under routine follow-up with the department of otorhinolaryngology and internal medicine. The patient had no other complaints at his fourth-month follow-up, and his diplopia was resolved.

## Discussion

Mucormycosis denotes a cluster of life-threatening infections caused by fungi of the order Mucorales of the subphylum Mucoromycotina. The Mucorales are ubiquitous in our environment, leading to constant exposure [7]. Mucormycosis is a rare disease with a prevalence of 0.005 to 1.7 per million in the general population. However, in India, the incidence of mucormycosis is 80 times higher than that reported in other parts of the world [4]. The significantly higher prevalence of COVID-19-induced mucormycosis in India may be multifactorial, including the role of poorly controlled diabetes, excessive use of corticosteroids and antibiotics, and environmental exposure. The hot and humid environment of India may have promoted the growth of Mucorales species [8].

The fungal spores enter the body through direct inhalation, ingestion of tainted food, or skin abrasions resulting in rhino-orbital-cerebral disease, pulmonary mucormycosis, cutaneous/wound infections, or gastrointestinal disease. Rhino-orbital-cerebral mucormycosis (ROCM) is the most commonly encountered presentation in India [7,9]. The nasal turbinates are initially infected in ROCM. In an immunocompromised host with impaired phagocytosis or ciliary clearance, the spores germinate into hyphae, and their angioinvasion leads to infarction, necrosis, and contiguous spread in the surrounding tissues [7,9]. The initial presentation is diverse and includes pain, nasal stuffiness, nasal discharge (bloody, black, purulent, or mucoid), epistaxis, edema (facial, periocular, or paranasal), discoloration (skin or the palate), dysarthria, visual impairment, and headaches [10].

Potential sequelae of intracranial spread are cavernous sinus or sagittal sinus thrombosis, carotid occlusion, cerebral infarction, intracranial aneurysms, intracranial hemorrhage, and cerebral abscesses [2].

In our opinion, these are the first two cases of mucormycotic aneurysms of the ICA in a post-COVID-19 scenario. Even with appropriate therapy, the mortality of ROCM is approximately 50% [11].

IV liposomal amphotericin B (10 mg/kg/day) is widely regarded as the antifungal agent of choice for ROCM, especially with central nervous system involvement. Early resection or debridement is the key to disease control as amphotericin cannot penetrate the necrotic tissue. Isavuconazole and posaconazole are considered second-line agents or salvage options once the disease is stabilized. They can also act as add-ins in case of extensive disease. The role of hyperbaric oxygen therapy or triple therapy (a combination of echinocandins, lipid polyene, and an azole) needs further validation [7,9].

A recent review of the literature found that 36.66% of all reported cases of post-COVID-19 mucormycosis were from India and that ROCM was the most common form of presentation. Diabetic patients had higher mortality than non-diabetics (49% vs. 30%). Good glycemic control and low-dose corticosteroid therapy dramatically reduced the incidence of post-COVID-19 mucormycosis [4].

Azar et al. observed that fungal mycotic aneurysms of the ICA had a mortality of 66%, with death occurring between 10 days and 16 months after the diagnosis. The causes of death were acute aneurysmal rupture, chronic complications related to the aneurysm rupture, and procedure-related complications. Endovascular therapy (ET) is increasingly preferred over surgery due to its lower morbidity and comparable mortality. Moreover, the open surgical approach requires favorable anatomical access and good surgical candidacy, which is not always possible [5].

Due to the recent advances in ET, numerous modalities of treatment approaches have become available, such as coiling of the aneurysm and occlusion of the parent artery. Novel techniques such as flow diversion and stent-assisted or balloon-assisted coiling are employed for aneurysms with a wide neck or complex anatomy when preservation of the distal circulation is prioritized [5].

A retrospective study by Chapot et al. found that the two-year post-ET survival rate of cerebral mycotic aneurysms was 100% [12]. A two-year post-ET survival rate of 82.2% for mycotic aortic aneurysms was observed in a review by Kan et al. [13].

A few concerns regarding ET compared to the open surgical approach include coil placement in an infected site and suboptimal source control. Although this might be true in the case of severe immunosuppression, the literature pertaining to this debate is sparse and more research is needed.

We chose to occlude the ICA along with the aneurysm in Case 1 because the ICA in and around the aneurysmal segment was diseased and preserving it was not our goal. Moreover, because the patient was actively bleeding, immediate coiling and parent artery occlusion was the safer choice [5,14]. In Case 2, the aneurysm was large and sporting a wide neck which justified the use of a flow diverter. Though flow diversion is superior to coiling, it cannot immediately prevent rupture. Although the patient was not actively bleeding at the time of surgery, based on his history of aneurysmal bleeding, flow-diverter-assisted coiling was preferred to preserve the distal perfusion [14,15].

The coiling of an aneurysm promotes thrombosis by reducing the blood flow into the aneurysm, lowering the velocity, increasing the residence time of blood within the aneurysmal space, and decreasing the aneurysmal wall shear stress. The complete healing of an aneurysm might take more than six months [5,16]. Flow diversion covers the neck of the aneurysm, and its mesh reduces the blood flow into the aneurysm. The stasis of flow within the aneurysm promotes thrombosis. The flow diverter also provides better scaffolding for neo-endothelialization across the aneurysm neck compared to coiling alone [14,17,18].

## Conclusions

The rapid surge of cases during the second wave of the pandemic highlighted various possible manifestations of COVID-19, particularly immune dysregulation and susceptibility to secondary infections. India reported the most cases of post-COVID-19 mucormycosis and its devastating sequelae. A high index of suspicion is required for the early diagnosis and treatment, which improves health outcomes. These disastrous consequences can be mitigated by curbing the rampant misuse of antibiotics, immunomodulators, and optimal glycemic control. Physicians should be aware of such rare and unique presentations for better tackling the complex manifestations of COVID-19.
